# A Stable Tetraphenylethylene-Based Charge-Assisted Hydrogen-Bonded Organic Framework for Turn-On Fluorescence Sensing of Al^3+^ Ions

**DOI:** 10.3390/molecules30244725

**Published:** 2025-12-10

**Authors:** Yingjia Deng, Yijin Wang, Xiangyu Gao, Yunke Jin, Jiabao Liu, Guanglai Mo, Yixuan Guo, Lanlu Lu, Peng Li

**Affiliations:** 1 State Key Laboratory of Porous Materials for Separation and Conversion, Shanghai Key Laboratory of Molecular Catalysis and Innovative Materials, Department of Chemistry, College of Smart Materials and Future Energy, Fudan University, 2005 Songhu Road, Shanghai 200438, China; 2 School of Chemical Engineering and Technology, Hebei University of Technology, Xiping Dao 5340, Beichen District, Tianjin 300401, China; 3 National Facility for Protein Science in Shanghai, Shanghai Advanced Research Institute, Chinese Academy of Sciences, Shanghai 201210, China

**Keywords:** charge-assisted hydrogen-bonded organic framework, stimuli-responsive fluorescence, Al^3+^ sensing, tetraphenylethylene (TPE)

## Abstract

The development of stable and sensitive fluorescent sensors for metal ion detection remains a challenge in materials chemistry. Although hydrogen-bonded organic frameworks (HOFs) have shown great potential in luminescent applications, their practical use is often limited by structural instability. In this work, we present a novel charge-assisted HOF, termed FDU-HOF-21 ([H(NH_2_Bpy)]_2_(TPE)), constructed from a tetraphenylethylene (TPE)-based carboxylic acid ligand (H_4_TCPE) and 2,2′-bipyridine-5,5′-diamine (NH_2_Bpy). Single-crystal X-ray diffraction (SCXRD) reveals a stable three-dimensional framework stabilized by an extensive hydrogen-bonding network and reinforced by charge-assisted hydrogen bonds (CAHBs), and it exhibits exceptional stability across various solvents and pH conditions. Moreover, FDU-HOF-21 serves as a highly sensitive and selective fluorescent turn-on sensor for Al^3+^ ions, with a lowest limit of detection (LOD) of 1.7 × 10^−6^ M. Characterization and time-dependent density functional theory (TDDFT) calculations reveal that the fluorescence enhancement originates from the suppression of non-radiative decay likely due to the reduction in intermolecular charge transfer (Inter-CT) during the emission process, coupled with the restricted intramolecular rotation upon Al^3+^ chelation.

## 1. Introduction

Fluorescence sensing has become an indispensable analytical technique in fields such as environmental monitoring [1,2], bioimaging [3,4], anti-counterfeiting [5,6], and medical diagnostics [7,8], owing to its outstanding advantages of high resolution, superior sensitivity, excellent selectivity, rapid response, and potential for visual detection. In particular, the use of fluorescent sensors for specific ion recognition represents a key strategy for monitoring water pollution and metal ion homeostasis in biological systems [9,10,11,12]. In recent years, porous materials such as zeolites, metal organic frameworks (MOFs), and covalent organic frameworks (COFs) have been widely investigated for fluorescence sensing applications [13,14,15]. These materials possess high specific surface areas, tunable pore structures, and ease of functionalization [16,17], enabling the incorporation of specific recognition sites for guest molecules or ions, which facilitates highly efficient and selective sensing of targets such as explosives [18,19], organic pollutants [20,21], quantification of humidity [22] and various metal ions [23,24,25].

As an emerging type of porous crystalline material, hydrogen-bonded organic frameworks (HOFs) have attracted growing interest owing to their unique advantages, including mild synthesis conditions, solution processability, flexibility, high crystallinity and high biocompatibility [17,26,27,28,29]. Moreover, as their building units can be rationally designed as fluorophores, HOFs, which are composed primarily of organic components, exhibit great potential for luminescent and sensing applications [30]. To date, numerous HOFs have been developed for applications in areas such as cellular imaging [31], chemical sensing [27,32,33,34], stimuli-responsive sensing [28,35] and anti-counterfeiting [36]. However, most HOFs are constructed from a single type of building unit and relatively weak hydrogen bonds, which leads to poor structural stability after desolvation, a narrow pH stability range, and structural simplicity [17]. The introduction of electrostatic interactions has been recognized as an effective strategy to enhance the stability of HOF structures [37,38,39]. In recent years, a variety of charge-assisted HOFs based on different functional groups have been reported, such as guanidium-sulfonate [40], ammonium-sulfonate [41], pyridinium-carboxylate [42,43], amidinium-carboxylate [44] or ammonium-carboxylate [45,46,47,48]. Furthermore, the multicomponent composition of charge-assisted HOFs not only favors the exploration of diverse ligands for constructing novel architectures but also facilitates the introduction of additional functional groups as potential recognition sites, thereby expanding their application prospects. Nevertheless, the application of both conventional and charge-assisted HOFs in the fluorescence sensing of metal ions has rarely been explored.

Tetraphenylethylene (TPE) derivatives, which exhibit aggregation-induced emission (AIE), have been widely employed in the construction of luminescent HOFs [30,31,43,49]. Herein, we report a novel charge-assisted HOF, denoted as FDU-HOF-21, constructed from a TPE-derived carboxylic acid (1,1,2,2-Tetra(4-carboxylphenyl) ethylene, H_4_TCPE) and a nitrogen-containing ligand (2,2′-bipyridine-5,5′-diamine, NH_2_Bpy). The charge-assisted assembly not only enhances the stability of the framework but also provides additional coordination sites for selective sensing. Moreover, the introduced functional groups form donor–acceptor (D–A) pairs interconnected via hydrogen-bonding networks, which serve as an efficient pathway for intermolecular charge transfer (Inter-CT), thereby endowing the material with novel fluorescent properties. FDU-HOF-21 exhibits excellent stability and a unique fluorescence enhancement response toward Al^3+^ ions. The fluorescence enhancement mechanism was systematically investigated through spectroscopic characterization and time-dependent density functional theory (TDDFT) calculations. It was revealed that coordination with Al^3+^ suppressed the original Inter-CT process in the FDU-HOF-21. Concurrently, the chelation with Al^3+^ effectively restricts intramolecular rotation, thereby suppressing non-radiative decay and ultimately leading to the observed fluorescence enhancement.

## 2. Results and Discussion

### 2.1. Synthesis, Structure, and Characterizations of FDU-HOF-21

**Figure 1:**
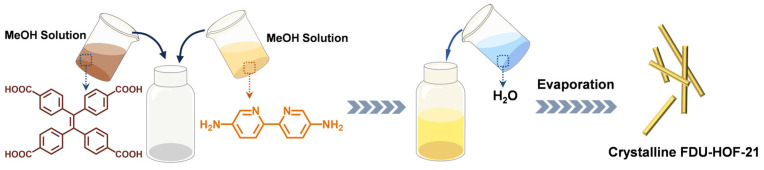
Synthetic process of FDU-HOF-21.

**Figure 2:**
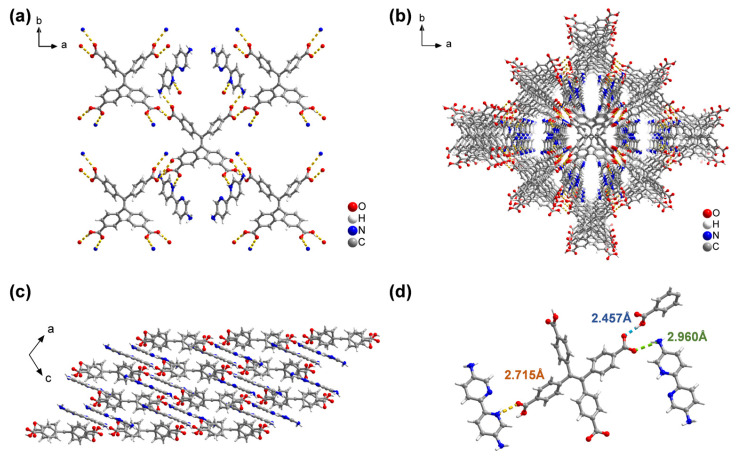
Crystal structure of FDU-HOF-21. (**a**) View of the connection of adjacent building blocks; representation of the framework along (**b**) c-axis and (**c**) b-axis; (**d**) different types of hydrogen bonds in HOF (Color codes: C, gray; H, white; O, red; N, blue; H-bond, dotted lines).

The H_4_TCPE and NH_2_Bpy linkers were purchased from commercial sources. Single crystals of FDU-HOF-21 can be obtained by mixing their methanol solutions with water followed by slow solvent evaporation at room temperature over 48 h (Figure 1). Single-crystal X-ray diffraction (SCXRD) analysis (Table S1) reveals FDU-HOF-21 in a monoclinic *C*2/*c* space group (unit cell parameters: *a* = 24.1153(18) Å, *b* = 18.1561(13) Å, *c* = 11.1232(7) Å, *α* = *γ* = 90°, *β* = 110.316(2)°), and the molecular formula was determined to be [H(NH_2_Bpy)]_2_(TPE) (C_30_H_18_O_8_, 2(C_10_H_11_N_4_)). As shown in Figure 2, in FDU-HOF-21, two oxygen atoms from each H_4_TCPE molecule forming N···H−O hydrogen bonds with nitrogen atoms of the amino groups on NH_2_Bpy ligands (2.960 Å), while two additional oxygen atoms establish O−H···N hydrogen bonds with pyridinic nitrogen atoms (2.715 Å). The H_4_TCPE frameworks are interconnected through intermolecular dimer (O−H···O, 2.457 Å) with adjacent units, forming a stable three-dimensional HOF. The above hydrogen bonds extend along the a- and b-axes, generating one-dimensional channels parallel to the c-axis direction. Notably, protonation occurs at nitrogen atoms of NH_2_Bpy ligands, which form charge-assisted hydrogen bonds (CAHBs) with carboxylate ions. The structural analysis of the material was performed by using the open source software tool Zeo++ (version 0.3) [50] (Figure S2 and Table S2). The accessible volume fraction of FDU-HOF-21 was determined to be 37.6% (accessible pore volume of 1718.73 Å^3^). The global cavity diameter (maximum included sphere diameters) is 3.713 Å, the pore limiting diameter (largest free sphere diameter) is 2.508 Å, and the largest cavity diameter is 3.706 Å. These results verified the porosity of FDU-HOF-21, establishing it as a porous HOF material.

**Figure 3:**
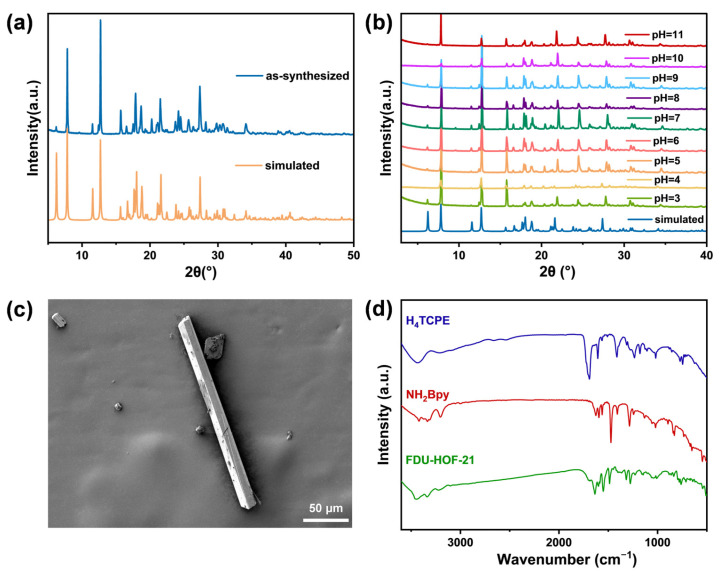
(**a**) PXRD patterns of FDU-HOF-21; (**b**) PXRD patterns of FDU-HOF-21 after treatment with aqueous solutions at different pH; (**c**) SEM image of FDU-HOF-21; (**d**) FT-IR spectra of H_4_TCPE, NH_2_Bpy and FDU-HOF-21.

The crystallinity and morphology of the synthesized FDU-HOF-21 were evaluated via powder X-ray diffraction (PXRD) and scanning electron microscopy (SEM). The PXRD patterns of FDU-HOF-21 were closely matched with the simulated one (Figure 3a) from single-crystal data. In addition, FDU-HOF-21 also maintained its crystallinity under aggressive pH conditions from 3 to 11 (Figure 3b) and displayed good stability in common solvents including acetone, cyclohexane, acetonitrile and water, as well as structural integrity in ethanol (albeit with reduced crystallinity) (Figure S3). HOF materials that possess such a wide pH tolerance are rarely reported, highlighting its robustness for wide application and potential for functional deployment. FDU-HOF-21 was observed to exhibit a yellow rod-shaped morphology under both SEM (Figure 3c and Figure S4) and optical microscopy (Figure S5). Furthermore, the ^1^H nuclear magnetic resonance (NMR) demonstrated a 1:2 stoichiometric ratio between the two ligands H_4_TCPE and NH_2_Bpy in FDU-HOF-21 (Figures S6 and S7), which corresponds to its molecular formula. Fourier transform infrared (FT-IR) spectroscopy (Figure 3d) shows the disappearance of the carboxyl group stretching vibration peak at 1700 cm^−1^ in the H_4_TCPE ligands and the appearance of new carboxylate stretching vibration peak at 1550 cm^−1^ in the HOF [51]. Interaction region indicator (IRI) analysis was conducted to characterize the chemical bonding and weak interactions in the structure. As indicated by the solid arrows in Figure S8, the protonated nitrogen atoms of the pyridine and amino groups form CAHBs with carboxylic acid sites in H_4_TCPE in FDU-HOF-21. Density functional theory (DFT) calculations further confirm the presence of CAHBs between the amino/pyridine groups and carboxylic acids. The binding energies of the N–H···O hydrogen bonds were calculated to be 42.3 kJ/mol (pyridine) and 40.9 kJ/mol (amino) (Figure S9), which are higher than those of conventional hydrogen bonds, thus contributing to the superior stability of the framework [26,52,53]. These results directly establish FDU-HOF-21 as a charge-assisted HOF.

### 2.2. Fluorescence Response of FDU-HOF-21 to Al^3+^

**Figure 4:**
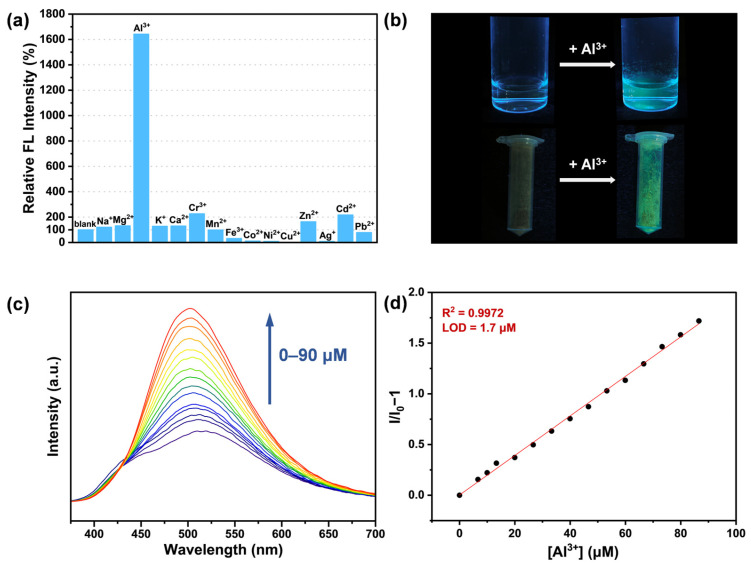
(**a**) Fluorescence (FL) intensity changes of FDU-HOF-21 aqueous dispersion in response to various ions at λ_ex_ = 330 nm; (**b**) photographs of FDU-HOF-21 aqueous dispersion and powders after treated with Al^3+^ (under ultraviolet radiation light); (**c**) fluorescence spectra of FDU-HOF-21 treated with different concentrations of Al^3+^ ions at λ_ex_ = 330 nm; (**d**) linear curve of FDU-HOF-21 with different concentrations of Al^3+^ ions.

Given the excellent stability of FDU-HOF-21 in aqueous solution and its adaptability to acidic and alkaline environments, we explore its fluorescence sensing capability toward metal ions in water. Therefore, fluorescence sensing abilities of the FDU-HOF-21 towards various common metal ions (Na^+^, K^+^, Ag^+^, Mg^2+^, Ca^2+^, Mn^2+^, Co^2+^, Ni^2+^, Cu^2+^, Zn^2+^, Cd^2+^, Pb^2+^, Al^3+^, Cr^3+^, Fe^3+^, all metal ions were used in the form of their chloride salts, with the exception of silver acetate) were investigated under excitation conditions of 330 nm and 298 K. The fluorescence emission spectra revealed that after the addition of Al^3+^ (1 mM), the emission peak of FDU-HOF-21 exhibited a blue shift from 510 nm to 500 nm, together with a more than 15-fold enhancement in fluorescence intensity—significantly greater than changes induced by other metal ions (Figure 4a and Figure S10). Notably, solid powder samples of FDU-HOF-21 treated with Al^3+^ solution also exhibited remarkable fluorescence enhancement (Figure 4b). Subsequently, we further explored the fluorescence response of FDU-HOF-21 to different concentrations of Al^3+^. The fluorescence intensity gradually increases while adding Al^3+^ (Figure 4c). A calibration curve of FDU-HOF-21 towards Al^3+^ exhibits good linearity (y = 0.1949 x + 0.0024, *R*^2^ = 0.9972) within the concentration range of 0–90 μM (Figure 4d). The limit of detection (LOD) of FDU-HOF-21 for Al^3+^ in aqueous solution was calculated to be 1.7 μM based on 3*σ*/*S*. In addition to high sensitivity and selectivity, the response of FDU-HOF-21 to Al^3+^ was instantaneous (Figure S11), and the fluorescence intensity remained largely stable over tens of minutes.

### 2.3. Sensing Mechanism Exploration

**Figure 5:**
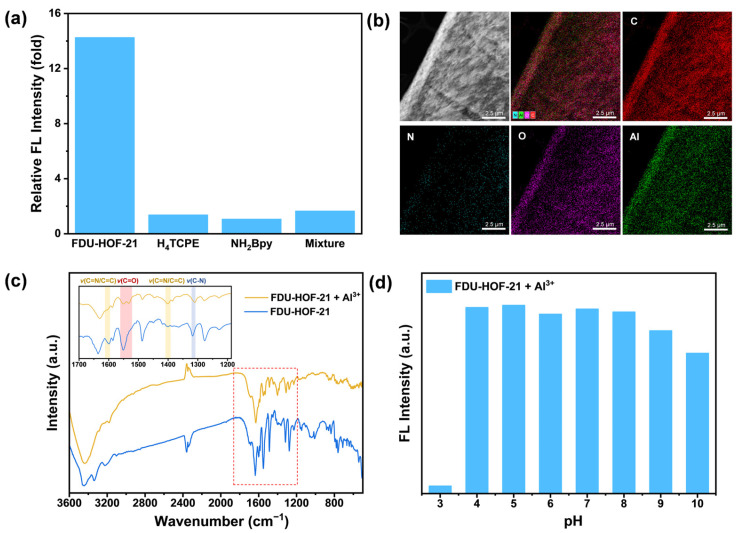
(**a**) Fluorescence enhancement of FDU-HOF-21, H_4_TCPE, NH_2_Bpy and their powder mixture upon adding Al^3+^ ions; (**b**) scanning transmission election microscope-high angle annular dark-field (STEM-HAADF) image of FDU-HOF-21 binding Al^3+^ and its EDS elemental mapping images for C, N, O, Al; (**c**) FT-IR spectra of FDU-HOF-21 before and after interaction with Al^3+^ ions; (**d**) fluorescence intensity changes of FDU-HOF-21 after addition of Al^3+^ under various pH conditions.

To gain deeper insights into the interaction mechanism between FDU-HOF-21 and Al^3+^, systematic investigations were conducted. Compared to the fluorescence spectra of the amorphous ligand molecules H_4_TCPE and NH_2_Bpy as well as their powder mixture, we observed that the enhanced fluorescence response to Al^3+^ occurs only in FDU-HOF-21, which possesses a periodically ordered structure (Figure 5a and Figure S12). This indicates that the Al^3+^ recognition capability of FDU-HOF-21 originates from cooperative interactions between its two constituent components. As revealed by Ultraviolet–Visible (UV-Vis) spectra (Figure S13), the absorption bands of FDU-HOF-21 shifts from 428 to 442 nm and a new absorption band emerged at 690 nm after Al^3+^ coordination. Energy-dispersive spectroscopy (EDS) mapping coupled with scanning transmission electron microscopy (STEM) and SEM were employed to analyze the elemental composition. As shown in Figure 5b and Figure S14, aluminum was uniformly distributed throughout FDU-HOF-21, indicating a definite interaction between FDU-HOF-21 and Al^3+^. In the FT-IR spectra (Figure 5c), the carboxylate stretching vibration peaks at 1550 cm^−1^ and pyridine C=N/C=C stretching vibration peaks at 1600 cm^−1^ and 1400 cm^−1^ exhibited alterations in peak morphology after the interaction of FDU-HOF-21 and Al^3+^. Additionally, the pyridine ring C-N stretching vibration displayed a shift from 1317 cm^−1^ to 1310 cm^−1^ upon Al^3+^ binding [54,55]. These shifts indicated the presence of Al–N and Al–O interactions during the response process. Moreover, an enhanced hydroxyl band can be observed around 3500 cm^−1^, suggesting the coordination between water and the Al^3+^ centers within the structure. The electrostatic potential (ESP) analysis within the pores results (Figure S15) indicate that the unprotonated nitrogen atom in NH_2_BPY and the adjacent carboxyl group exhibit a relatively low negative potential. This favors electrostatic attraction with Al^3+^ and suggests these sites are likely the binding locations for aluminum in FDU-HOF-21.

The fluorescence intensity of FDU-HOF-21 in the presence and absence of Al^3+^ was tested under various pH conditions. The experimental results indicate that the fluorescence response of FDU-HOF-21 toward Al^3+^ remained relatively stable within the pH range of 4–8 (Figure 5d). The decrease in fluorescence intensity at higher pH values is likely attributable to the formation of Al-(OH)_3_ under alkaline conditions, thereby confirming the specific recognition of aluminum ions by FDU-HOF-21. Notably, the addition of Al^3+^ to FDU-HOF-21 at pH = 3 does not lead to significant fluorescence enhancement. This phenomenon may originate from the protonation of recognition sites (especially carboxylate) in FDU-HOF-21 due to increased H^+^ concentration, thereby limiting the binding process between Al^3+^ and these sites. These observations further support the interaction between Al and O in the fluorescence response process under optimized pH conditions. To investigate the structural transformation, PXRD measurements were conducted on FDU-HOF-21 both before and after treatment with Al^3+^. The PXRD patterns showed no appreciable difference (Figure S16), demonstrating the robustness of the crystal structure.

**Figure 6:**
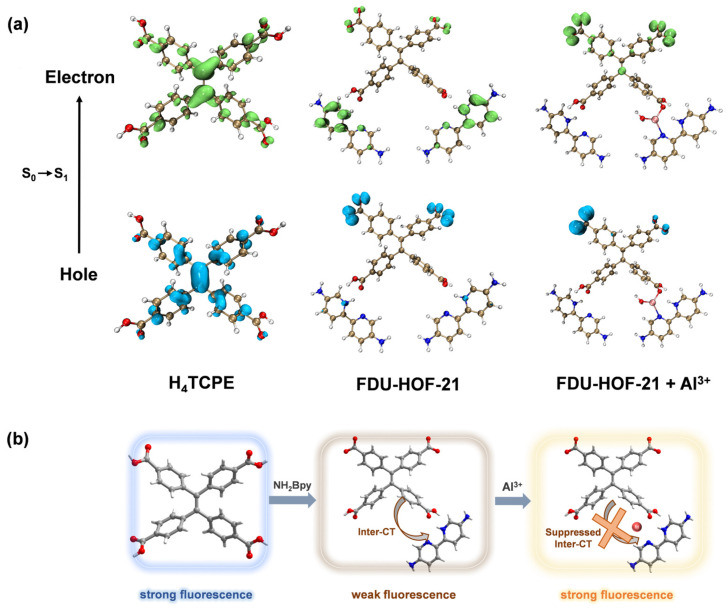
(**a**) TDDFT calculations and S_0_ → S_1_ electron–hole calculations for H_4_TCPE, FDU-HOF-21 and FDU-HOF-21+Al^3+^ (Color codes: electron, green; hole, blue); (**b**) proposed fluorescence mechanism for the formation of FDU-HOF-21 and its response to Al^3+^ ions.

The H_4_TCPE molecule, which exhibits AIE [56] characteristic, displays intense blue fluorescence. However, a pronounced fluorescence quenching and red shift was observed upon the formation of a HOF with NH_2_Bpy through CAHBs (Figure S12). We attribute this phenomenon to an efficient Inter-CT process, which is facilitated by a well-defined D–A structure assembled and stabilized within the hydrogen-bonded network. We have employed TDDFT calculations to investigate the fluorescence quenching mechanism of FDU-HOF-21. The hole-electron analysis reveals that in the FDU-HOF-21 ligand, both holes (blue) and electrons (green) were primarily localized on the TPE unit. In contrast, in FDU-HOF-21, the “hole” is primarily localized on the TPE unit of the backbone, while the “electron” is distributed over the electron-accepting NH_2_Bpy unit (Figure 6a). This distinct orbital separation provides direct evidence for an effective push–pull electronic system established in the HOF. This electronic distribution corresponds to a strong Inter-CT character for the S_0_ → S_1_ transition, which is consistent with the observed weak fluorescence intensity of the material [5,57,58].

An increase in the fluorescence lifetime of the material was observed (Table S4 and Figure S17) after the formation of the FDU-HOF-21+Al^3+^ complex, indicating suppression of the non-radiative transition process. We also observed that a new excitation peak emerged in the excitation spectrum of the complex during the binding with Al^3+^ (Figure S18), which exhibited a more pronounced enhancement with increasing concentration compared to the original excitation peak. This new excitation peak corresponds to a higher-energy excited state and does not lead to a shift in the emission maximum. The coordination of Al^3+^ ions with nitrogen and oxygen atoms in FDU-HOF-21 likely introduces a new electronic state. TDDFT calculations reveal that upon coordination with Al^3+^, both the “hole” and “electron” are found to be predominantly located on the TPE unit. These results indicate that the original Inter-CT process is significantly suppressed after Al^3+^ binding, which effectively reduces the non-radiative decay pathway and accounts for the observed turn-on fluorescence response [58]. Furthermore, the chelation of Al^3+^ ions within the HOF structure effectively restricts the rotational freedom of the ligands, the rigidity of the organic ligand further increased [59], thereby limiting intramolecular motions and suppressing non-radiative decay [60,61]. These mechanism account for the observed fluorescence enhancement and extended lifetime.

## 3. Materials and Methods

### 3.1. Materials

H_4_TCPE, NH_2_Bpy were supplied by Yanshen Technology Co., Ltd. (Changchun, China). Ethanol, Tetrahydrofuran (THF), isopropanol, magnesium chloride (MgCl_2_), chromic chloride (CrCl_3_), nickel chloride (NiCl_2_), cobalt chloride (CoCl_2_), manganese chloride (MnCl_2_) and silver acetate were supplied by Adamas-Beta Reagent Co., Ltd. (Shanghai, China). Iron chloride (FeCl_3_) was supplied by Sigma-Aldrich Trading Co., Ltd (Shanghai, China). Lead chloride (PbCl_2_) and aluminum chloride (AlCl_3_) were supplied by Shanghai Aladdin Biochemical Technology Co., Ltd. (Shanghai, China). Copper chloride (CuCl_2_), zinc chloride (ZnCl_2_), potassium chloride (KCl), sodium hydroxide (NaOH), hexane, acetone, methanol and calcium chloride (CaCl_2_) were obtained from Shanghai Titan Scientific Co., Ltd. (Shanghai, China). Hydrochloric acid (HCl), sodium chloride (NaCl), cadmium chloride (CdCl_2_) and acetonitrile were supplied by Hushi Chemical Reagent Co., Ltd. (Shanghai, China). Dimethyl sulfoxide-*d_6_* (DMSO-*d*_6_) was supplied by Energy Chemical (Shanghai, China).

#### Synthesis of FDU-HOF-21 Single Crystalline

A mixture of 10 mg (0.02 mmol) H_4_TCPE and 7.5 mg (0.04 mmol) NH_2_Bpy was dissolved in 5 mL of methanol in a 20 mL glass vial, followed by the addition of 10 mL of water. The vial was allowed to stand under dry ambient conditions for slow evaporation at room temperature. Yellow rod-shaped crystals can be obtained after approximately 48 h, with a yield of around 40%. Powder samples can be synthesized by scaling up the procedure accordingly. After washing the precipitate with distilled water and acetone, and then dried at 60 °C for one day to obtain yellow crystalline powder.

### 3.2. Methods

#### 3.2.1. General Procedures

The morphology of the resulting crystal structures was characterized using a Hitachi Flex SEM 1000 Scanning Electron Microscope (Hitachi, Tokyo, Japan). EDS mapping images were obtained on a GeminiSEM 560 field emission scanning electron microscope (ZEISS, Oberkochen, Germany) and a field emission transmission electron microscope equipped with an energy dispersive spectrometer Tecnai G2 F20 S-Twin (FEI, Hillsboro, OR, USA). Solution ^1^H NMR spectra were collected by Bruker AVANCE III 400 MHz spectrometers (Bruker Corporation, Karlsruhe, Germany). The FT-IR spectra of the powder samples were recorded in the frequency range of 400–4000 cm^−1^ with a resolution of 4 cm^−1^ by accumulating 32 scans using a KBr disc method on a Thermo Fisher Nicolet iS10 spectrometer (Thermo Fisher Scientific, Waltham, MA, USA). Fluorescence spectra were obtained by Cytation 3 Multi-mode Microplat (BioTek, Winooski, VT, USA) and FLS1000 (Edinburgh Instruments, Livingstone, UK). The fluorescence lifetime was collected by FLS1000 (Edinburgh Instruments, Livingstone, UK). Solid UV–vis spectra were measured with a Lambda 650S UV–vis spectrophotometer (PERKIN ELMER, Waltham, MA, USA). PXRD patterns were recorded by Smartlab 9 kW X-ray diffractometer (Rigaku, Tokyo, Japan) equipped with a Cu rotating anode X-ray source.

#### 3.2.2. Single-Crystal X-Ray Diffraction

Single crystal diffraction data were measured at the BL17B1 High-throughput Protein Crystallography Beamline in Shanghai Synchrotron Radiation Facility (SSRF). The structure was solved and refined with the SHELXL (version 2017/1) soft package [62,63] structure solution program using Charge Flipping and refinement package using Least Squares minimization. Details of the crystal data and refinement data are listed in Table S1. The X-ray crystallographic coordinates for the single-crystal structures have been deposited at the Cambridge Crystallographic Data Centre (CCDC), under deposition numbers CCDC 2496998. The data can be obtained free of charge via www.ccdc.cam.ac.uk/structures (accessed on 21 October 2025) (or from the Cambridge Crystallographic Data Centre, 12 Union Road, Cambridge CB2 1EZ, U.K.)

#### 3.2.3. Topological Analyses of FDU-HOF-21

The pore space within the crystal structure was topologically analyzed using Zeo++ (version 0.3) [50], yielding key parameters including surface areas, pore volumes, and pore diameters. These following pore descriptors were calculated: Unit cell volume, density of the crystal, the diameter of the largest included sphere, the largest free sphere diameter, the largest included sphere along the free path, the simulated pore size distribution plot, accessible surface area, accessible pore volumes and fraction. The pore size distribution was simulated and visualized using Materials Studio 2019.

#### 3.2.4. Fluorescence Sensing

A stable suspension was prepared by sonicating finely ground FDU-HOF-21 powder (1 mg) dispersed in 10 mL of deionized water for 30 min. Aqueous solutions (5 mM) of various metal ions (Na^+^, K^+^, Ag^+^, Mg^2+^, Ca^2+^, Mn^2+^, Co^2+^, Ni^2+^, Cu^2+^, Zn^2+^, Cd^2+^, Pb^2+^, Al^3+^, Cr^3+^, Fe^3+^) were prepared using deionized water. Then, 50 µL of each metal ion solution (or deionized water as control) was added to 200 µL of the FDU-HOF-21 suspension. Fluorescence spectra of the mixtures were recorded at room temperature under an excitation wavelength of 330 nm. The Al-treated solid sample was prepared by immersing the powder in Al^3+^ solution, followed by centrifugation, washing and drying.

To confirm the linear relationship between the fluorescence intensity and concentration, 10 mM Al^3+^ solution (1–30 µL) was gradually added to 3 mL of the FDU-HOF-21 suspension, and fluorescence spectra were detected under 330 nm excitation. The results indicated that the fluorescence intensity increased linearly with Al^3+^ concentration.

#### 3.2.5. The Double-Exponential Fitting Function

The fluorescence lifetime of FDU-HOF-21 was characterized by steady-state and transient fluorescence spectroscopy under an excitation of 350 nm at room temperature. The decay profiles were fitted to a bi-exponential function, expressed as:

(1) $$I=A_{1}\exp\left(-\frac{t}{\tau_{1}}\right)+\;A_{2}\exp\left(-\frac{t}{\tau_{2}}\right)$$

where *A*_1_ and *A*_2_ denote the amplitude factors, *t* represents time, and *τ*_1_ and *τ*_2_ correspond to the decay lifetimes. The average fluorescence lifetime (*τ*_ave_) was subsequently determined using the following equation:

(2) $$\tau_{ave}=\frac{A_{1}\tau_{1}^{2}+A_{2}\tau_{2}^{2}}{A_{1}\tau_{1}+A_{2}\tau_{2}}$$

#### 3.2.6. DFT Calculations

All calculations were carried out using the quantum chemistry software package Gaussian 09 [64]. All geometry optimization was based on the single crystal structures. The B3LYP functional [65,66,67] combined with Grimme’s D3 dispersion model and Becke-Johnson damping [66] was adopted, and the molecular structures were optimized and the vibrational frequencies were calculated using the 6-31G’(d, p) basis set [68,69]. For the hole-electron analysis [70], we used Multiwfn version 3.8 [70,71,72,73]. The absorption properties were obtained using TDDFT with the B3LYP functional at the same basis set level. Additionally, the hydrogen bond energy was calculated using the bond critical point (BCP) method [74]. The IRI analysis [75], was performed using the Multiwfn program. The analysis results were visualized using the VMD software (version 1.9.3) [76]. The geometry optimization of FDU-HOF-21 and its complexes with aluminum was conducted at the B3LYP functional and 6-311G’ (d, p) basis set level. Meanwhile, the solvent effect in aqueous solution was considered through the polarizable continuum model (PCM) implicit solvent model [27].

## 4. Conclusions

In summary, we have successfully constructed a novel charge-assisted hydrogen-bonded organic framework, FDU-HOF-21, based on a TPE-derived carboxylic acid H_4_TCPE and a nitrogen-containing ligand. The framework is stabilized by an extensive hydrogen-bonding network reinforced by CAHBs. This structural design endows FDU-HOF-21 with excellent chemical stability across a wide pH range and in various solvents, which is a property rarely achieved by conventional HOFs. More importantly, the introduce of nitrogen-containing ligand also provides additional coordination sites for selective sensing. FDU-HOF-21 functions as a highly sensitive and selective turn-on fluorescent sensor for Al^3+^ ions, with a LOD of 1.7 × 10^−6^ M. Through a combination of structural and spectroscopic characterization as well as TDDFT calculations, the fluorescence enhancement mechanism has been revealed: The hydrogen-bond-directed formation of the D–A structure in FDU-HOF-21 and the resulting intermolecular charge transfer, are identified as the primary cause of the fluorescence quenching compared to H_4_TCPE. After binding with Al^3+^ ions, the original intermolecular charge transfer process is reduced, suppressing non-radiative decay and resulting in significant fluorescence enhancement and extended emission lifetime. This work not only presents a stable charge-assisted HOF but also demonstrates its outstanding performance in fluorescence sensing, highlighting the potential of multicomponent assembly in the rational design of stable and functional porous materials. We will further focus on extending this strategy to design more functional charge-assisted HOFs and exploring their applications in stimuli-responsive sensing.

## Data Availability

The original contributions presented in the study are included in the article. Further inquiries can be directed to the corresponding author.

## Supplementary Materials

The following supporting information can be downloaded at: https://www.mdpi.com/article/10.3390/molecules30244725/s1, Figure S1. Local hydrogen bonding environments of FDU-HOF-21 molecules. Hydrogen bonds are indicated by dashed lines. Color code: O, red; N, blue; C, dark grey; H, white. Figure S2. (a–c) Pore distribution of the FDU-HOF-21; Figure S3. PXRD patterns of FDU-HOF-21 after treatment with different solvents for 24 h; Figure S4. SEM images of FDU-HOF-21; Figure S5. Microscopic optical photo for FDU-HOF-21; Figure S6. ^1^H NMR spectra of FDU-HOF-21, NH_2_Bpy and H_4_TCPE (400 MHz, DMSO-*d_6_*); Figure S7. ^1^H NMR spectra of FDU-HOF-21 and the corresponding integrations (400 MHz, DMSO-*d_6_*); Figure S8. IRI analysis for FDU-HOF-21: (a) carboxylic acid–pyridine and (b) carboxylic acid–amino interactions. Red, green, and blue areas denote strong repulsion, van der Waals interactions, and strong attractive forces (CAHBs), respectively; Figure S9. Calculation of the binding energies of the CAHBs; Figure S10. Fluorescence spectra of FDU-HOF-21 responded to different metal ions; Figure S11. Fluorescence intensity of FDU-HOF-21 versus time at 330 nm upon addition of Al^3+^; Figure S12. Fluorescence spectra of FDU-HOF-21, H_4_TCPE, NH_2_Bpy and H_4_TCPE-NH_2_Bpy mixture before and after responded to Al^3+^ ions; Figure S13. Solid-state UV-Vis spectra of FDU-HOF-21 before and after Al^3+^ binding; Figure S14. SEM-EDS elemental mapping images of FDU-HOF-21 for C, N, O, and Al (Top: before Al^3+^ binding; Bottom: after Al^3+^ binding); Figure S15. Electrostatic potentials mapped of FDU-HOF-21; Figure S16. PXRD patterns of FDU-HOF-21 before and after binding with aluminum ions; Figure S17. Time-resolved decay curves of FDU-HOF-21 before and after treatment by aluminum at λ_ex_ = 350 nm; Figure S18. Fluorescence excitation spectra of FDU-HOF-21 treated with different concentrations of Al^3+^ ions. Table S1. Crystallographic data of FDU-HOF-21; Table S2. The pore descriptors simulated by Zeo++; Table S3. Hydrogen bonds parameters in FDU-HOF-21; Table S4. The double exponential fitting parameters of time-resolved PL decay curves for FDU-HOF-21 and FDU-HOF-21+Al^3+^.
