# Supplementary material for: A Stable Tetraphenylethylene-Based Charge-Assisted Hydrogen-Bonded Organic Framework for Turn-On Fluorescence Sensing of Al3+ Ions
Source: Molecules. 2025 Dec 10;30(24):4725. doi: 10.3390/molecules30244725 (PMC12735657; doi:10.3390/molecules30244725)

checkCIF/PLATON report

Structure factors have been supplied for datablock(s) nh2bpy_0m_a

THIS REPORT IS FOR GUIDANCE ONLY. IF USED AS PART OF A REVIEW PROCEDURE FOR PUBLICATION, IT SHOULD NOT REPLACE THE EXPERTISE OF AN EXPERIENCED CRYSTALLOGRAPHIC REFEREE.

No syntax errors found.   CIF dictionary   Interpreting this report

Datablock: nh2bpy_0m_a

Bond precision: C-C = 0.0053 A   Wavelength=0.71073

Cell:	a=24.1153(18)	b=18.1561(13)	c=11.1232(7)
	alpha=90	beta=110.316(2)	gamma=90
Temperature:	100 K		

	Calculated	Reported
Volume	4567.2(6)	4567.2(6)
Space group	C 2/c	C 1 2/c 1
Hall group	-C 2yc	-C 2yc
Moiety formula	C30 H18 O8, 2(C10 H11 N4) [+ solvent]	C30 H18 O8, 2(C10 H11 N4)
Sum formula	C50 H40 N8 O8 [+ solvent]	C50 H40 N8 O8
Mr	880.90	880.90
Dx,g cm-3	1.281	1.281
Z	4	4
Mu (mm-1)	0.089	0.089
F000	1840.0	1840.0
F000'	1840.83	
h,k,lmax	29,21,13	29,21,12
Nref	4189	3875
Tmin,Tmax	0.989,0.991	0.664,0.746
Tmin'	0.956	

Correction method= # Reported T Limits: Tmin=0.664 Tmax=0.746
AbsCorr = NONE

Data completeness= 0.925 Theta(max)= 25.345

R(reflections)= 0.0966(3157) wR2(reflections)= 0.2030(3875)

S = 1.143 Npar= 283

The following ALERTS were generated. Each ALERT has the format
test-name_ALERT_alert-type_alert-level.
Click on the hyperlinks for more details of the test.

Alert level A

PLAT029_ALERT_3_A _diffrn_measured_fraction_theta_full value Low . 0.926 Why?

Author Response: This problem is caused by the fact that the synchrotron radiation sour

---

Alert level B

PLAT097_ALERT_2_B Large Reported Max. (Positive) Residual Density 0.83 eA-3

Author Response: This problem is caused by twin crystal.

```
PLAT911_ALERT_3_B Missing FCF Refl Between Thmin & STh/L=    0.600        287 Report
                 0 16  0,   1  9  0,   7  7  0,   8  4  0,  10  0  0,  10  4  0,
                18  4  0,  20  0  0,  25  1  0,  25  5  0, -25  7  1, -16  8  1,
               -14 10  1, -10  2  1,  -1  9  1,   1  9  1,   3  7  1,   3 15  1,
                 4 14  1,  10  2  1,  15  7  1,  18 12  1,  19  1  1,  22  6  1,
                -9  5  2,  -5  7  2,  -3  9  2,  -2  0  2,  -1  9  2,   4  6  2,
                     (  257 More Missing: see the .ckf listing file)
```

Author Response: This problem is caused by the fact that the synchrotron radiation sour

---

Alert level C

```
DIFMX02_ALERT_1_C  The maximum difference density is > 0.1*ZMAX*0.75
            The relevant atom site should be identified.
PLAT213_ALERT_2_C Atom C00F           has ADP max/min Ratio .....        3.3 oblate
PLAT213_ALERT_2_C Atom C008           has ADP max/min Ratio .....        3.3 prolat
PLAT213_ALERT_2_C Atom C00B           has ADP max/min Ratio .....        3.1 prolat
PLAT213_ALERT_2_C Atom C00Q           has ADP max/min Ratio .....        3.3 prolat
PLAT213_ALERT_2_C Atom C00J           has ADP max/min Ratio .....        3.2 prolat
PLAT213_ALERT_2_C Atom C00E           has ADP max/min Ratio .....        3.7 prolat
PLAT213_ALERT_2_C Atom C00D           has ADP max/min Ratio .....        3.2 prolat
PLAT250_ALERT_2_C Large U3/U1 Ratio for <U(i,j)> Tensor(Resd    1)       3.3 Note
PLAT250_ALERT_2_C Large U3/U1 Ratio for <U(i,j)> Tensor(Resd    2)       2.8 Note
PLAT340_ALERT_3_C Low Bond Precision on  C-C Bonds ...............    0.00531 Ang.
PLAT420_ALERT_2_C D-H Bond Without Acceptor  N006     --H00B     .     Please Check
PLAT420_ALERT_2_C D-H Bond Without Acceptor  N00K     --H00E     .     Please Check
```

```
PLAT906_ALERT_3_C Large K Value in the Analysis of Variance ......     11.463 Check
PLAT906_ALERT_3_C Large K Value in the Analysis of Variance ......      2.861 Check
PLAT975_ALERT_2_C Check Calcd Resid. Dens.  0.85Ang From O004    .       0.43 eA-3
PLAT977_ALERT_2_C Check Negative Difference Density on H003      .      -0.47 eA-3
```

Alert level G

```
PLAT002_ALERT_2_G Number of Distance or Angle Restraints on AtSite          3 Note
PLAT003_ALERT_2_G Number of Uiso or U(i,j)  Restrained non-H-Atoms          5 Report
PLAT007_ALERT_5_G Number of Unrefined Donor-H Atoms .............           5 Report
              H003    H00B    H00D    H00E    H00F
PLAT083_ALERT_2_G SHELXL Second Parameter in WGHT  Unusually Large      54.73 Why ?
PLAT128_ALERT_4_G Alternate Setting for Input Space-group     C2/c       I2/a Note
PLAT172_ALERT_4_G The CIF-Embedded .res File Contains DFIX Records          2 Report
PLAT186_ALERT_4_G The CIF-Embedded .res File Contains ISOR Records          5 Report
PLAT432_ALERT_2_G Short Inter X...Y Contact  C00D     ..C00N     .       3.13 Ang.
                                          1/2-x,1/2+y,1/2-z  =      4_555 Check
PLAT605_ALERT_4_G Largest Solvent Accessible VOID in the Structure        214 A**3
PLAT720_ALERT_4_G Number of Unusual/Non-Standard Labels ..........         54 Note
              O001    O002    O003    H003    O004    N005    N006    H00B
              H00D    C009    H009    C00I    H00I    C00F    C008    H008
              C00A    H00A    C007    C00C    H00C    C00G    H00G    C00B
              C00Q    H00Q    C00J    H00J    C00E    C00D    N00H    N00K
              H00E    H00F    C00L    C00M    C00N    H00N    C00O    H00O
              C00P    C00R    C00S    C00T    C00U    H00U    C00V    H00V
              C00W    C00X    H00X    C00Y    H00Y    H005
PLAT802_ALERT_4_G CIF Input Record(s) with more than 80 Characters          2 Info
PLAT860_ALERT_3_G Number of Least-Squares Restraints ............          32 Note
PLAT868_ALERT_4_G ALERTS Due to the Use of _smtbx_masks Suppressed          ! Info
PLAT912_ALERT_4_G Missing # of FCF Reflections Above STh/L=  0.600          2 Note
PLAT933_ALERT_2_G Number of HKL-OMIT Records in Embedded .res File          3 Note
               -8  2  7,  -3  9  5,  22  2  4,
PLAT969_ALERT_5_G The 'Henn et al.' R-Factor-gap value ..........       6.517 Note
              Predicted wR2: Based on SigI**2  3.11 or SHELX Weight 17.75
PLAT978_ALERT_2_G Number C-C Bonds with Positive Residual Density.          2 Info
```

---

  1 **ALERT level A** = Most likely a serious problem - resolve or explain
  2 **ALERT level B** = A potentially serious problem, consider carefully
 17 **ALERT level C** = Check. Ensure it is not caused by an omission or oversight
 17 **ALERT level G** = General information/check it is not something unexpected

```
  1 ALERT type 1 CIF construction/syntax error, inconsistent or missing data
 20 ALERT type 2 Indicator that the structure model may be wrong or deficient
  6 ALERT type 3 Indicator that the structure quality may be low
  8 ALERT type 4 Improvement, methodology, query or suggestion
  2 ALERT type 5 Informative message, check
```

It is advisable to attempt to resolve as many as possible of the alerts in all categories. Often the minor alerts point to easily fixed oversights, errors and omissions in your CIF or refinement strategy, so attention to these fine details can be worthwhile. It is up to the individual to critically assess their own results and, if necessary, seek expert advice.

PLATON version of 26/09/2025; check.def file version of 20/09/2025

Datablock nh2bpy_0m_a - ellipsoid plot

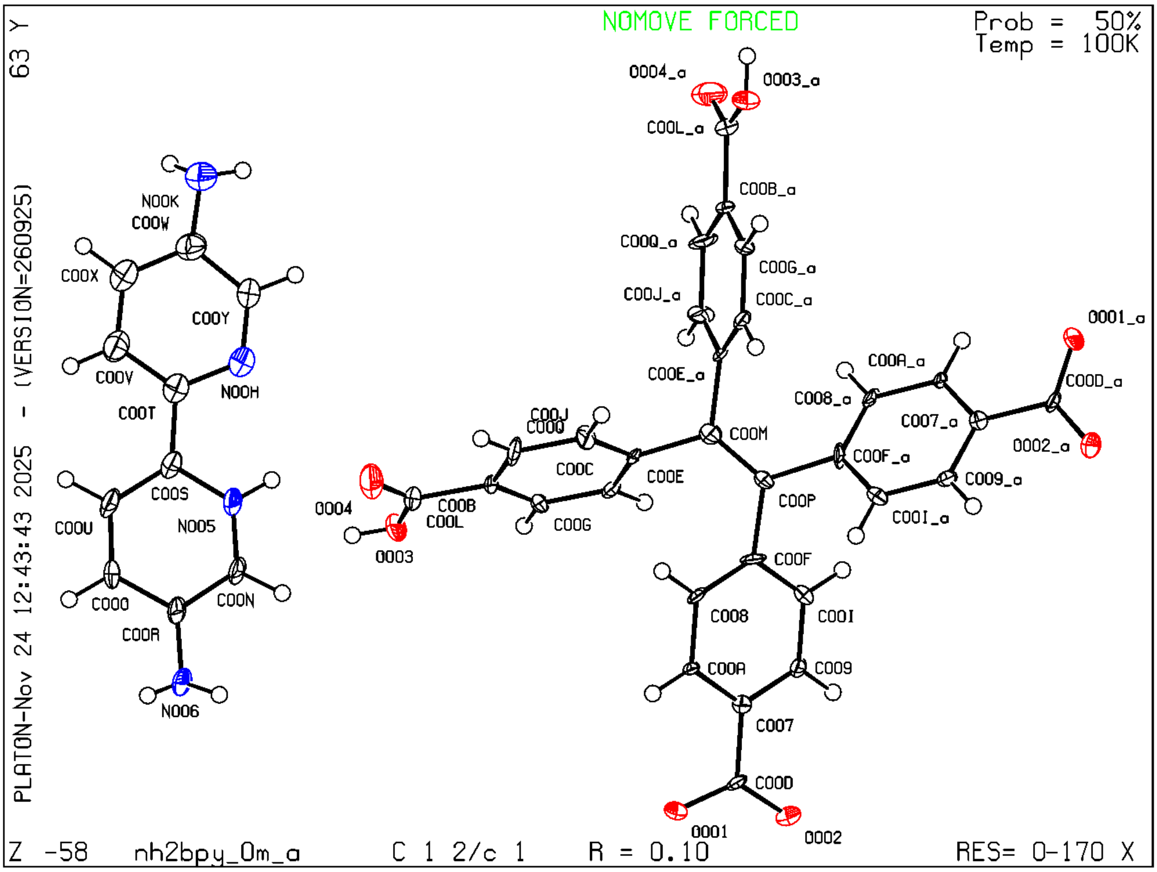

Supplement: Supplementary file 1 [file molecules-30-04725-s001.zip › checkcif FDU-HOF-21.pdf]
