# Supplementary material for: A Stable Tetraphenylethylene-Based Charge-Assisted Hydrogen-Bonded Organic Framework for Turn-On Fluorescence Sensing of Al3+ Ions
Source: Molecules. 2025 Dec 10;30(24):4725. doi: 10.3390/molecules30244725 (PMC12735657; doi:10.3390/molecules30244725)
Supplement: Supplementary file 1 [file molecules-30-04725-s001.zip › Deng et_al_Supporting_Information.pdf]

Supporting Information

A Stable Tetraphenylethylene-Based Charge-Assisted Hydrogen-Bonded Organic Framework for Turn-On Fluorescence Sensing of Al^{3+} Ions

Yingjia Deng[1], Yijin Wang[1], Xiangyu Gao[1], Yunke Jin[1], Jiabao Liu[2], Guanglai Mo[1], Yixuan Guo[1], Lanlu Lu[3] and Peng Li[1,*].

[1] State Key Laboratory of Porous Materials for Separation and Conversion, Shanghai Key Laboratory of Molecular Catalysis and Innovative Materials, Department of Chemistry, College of Smart Materials and Future Energy, Fudan University, 2005 Songhu Road, Shanghai, 200438, China.

[2] School of Chemical Engineering and Technology, Hebei University of Technology, Xiping Dao 5340, Beichen District, Tianjin 300401, China.

[3] National Facility for Protein Science in Shanghai, Shanghai Advanced Research Institute, Chinese Academy of Sciences, Shanghai 201210, China

* Correspondence: penglichem@fudan.edu.cn (P.L.)

Table S1. Crystallographic data of FDU-HOF-21.

Identification code	NH2BPy_0m_a
CCDC Number	2496998
Empirical formula	$C_{50}H_{40}N_8O_8$
Formula weight	880.9
Temperature/K	100
Crystal system	monoclinic
Space group	*C*2/*c*
a/Å	24.1153(18)
b/Å	18.1561(13)
c/Å	11.1232(7)
α/°	90
β/°	110.316(2)
γ/°	90
Volume/Å^3	4567.2(6)
Z	4
$\rho calc$ g/cm^3	1.281
μ/mm^{-1}	0.089
F(000)	1840
Crystal size/mm^3	0.5 × 0.1 × 0.1
Radiation	MoKα (λ = 0.71073)
2θ range for data collection/°	2.876 to 50.69
Index ranges	$-29 \le h \le 29$, $-21 \le k \le 21$, $-12 \le l \le 11$
Reflections collected	24684
Independent reflections	3878 [R_{int} = 0.0395, R_{sigma} = 0.0323]
Data/restraints/parameters	3875/32/283
Goodness-of-fit on F^2	1.143
Final R indexes [I ≥ 2σ (I)]	R_1 = 0.0966, ωR_2 = 0.1978
Final R indexes [all data]	R_1 = 0.1102, ωR_2 = 0.2030
Largest diff. peak/hole / e Å^{-3}	0.83/-0.54

Table S2. The pore descriptors simulated by Zeo++.

Parameters	Results
Unit cell volume	4567.22 Å^3
Crystal density	1.28406 g/cm^3
Diameter of the largest included sphere	3.713 Å
Diameter of the largest free sphere diameter	2.508 Å
The largest included sphere along the free path	3.706 Å
Accessible pore volumes	1718.73 Å^3
Accessible pore fraction	0. 37632

Table S3. Hydrogen bonds parameters in FDU-HOF-21.

Donor	H	Acceptor	d(D–H) / Å	d(H–A) / Å	d(D–A) / Å	D–H–A / °
O3	H3	O1	0.840	1.637	2.457	164.41
N6	HD	O2	0.817	2.188	2.960	157.64
N5	H5	O4	0.873	1.922	2.715	150.43

Table S4. The double exponential fitting parameters of time-resolved PL decay curves for FDU-HOF-21 and FDU-HOF-21+Al^{3+}.

	A_1(%)	τ_1(ns)	A_2(%)	τ_2(ns)	τ_{ave}(ns)
FDU-HOF-21	46.1389	0.2096	2.0977	1.2858	0.4443
FDU-HOF-21 + Al^{3+}	33.2318	0.4516	50.1536	2.1877	1.1848

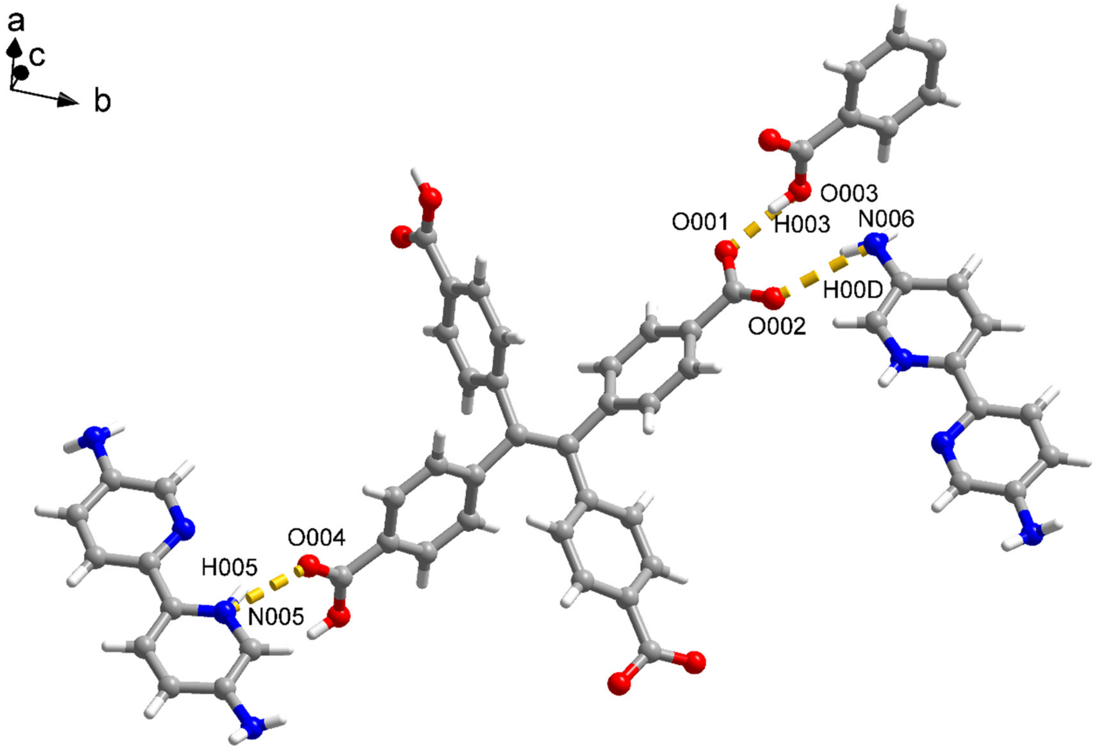

Figure S1. Local hydrogen bonding environments of FDU-HOF-21 molecules. Hydrogen bonds are indicated by dashed lines. Color code: O, red; N, blue; C, dark grey; H, white.

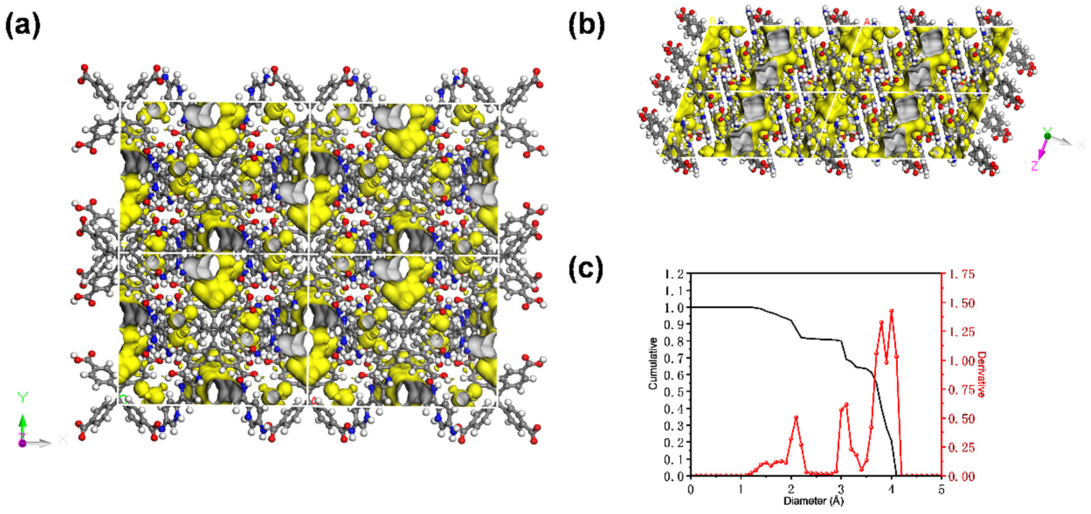

Figure S2. (a-c) Pore distribution of the FDU-HOF-21.

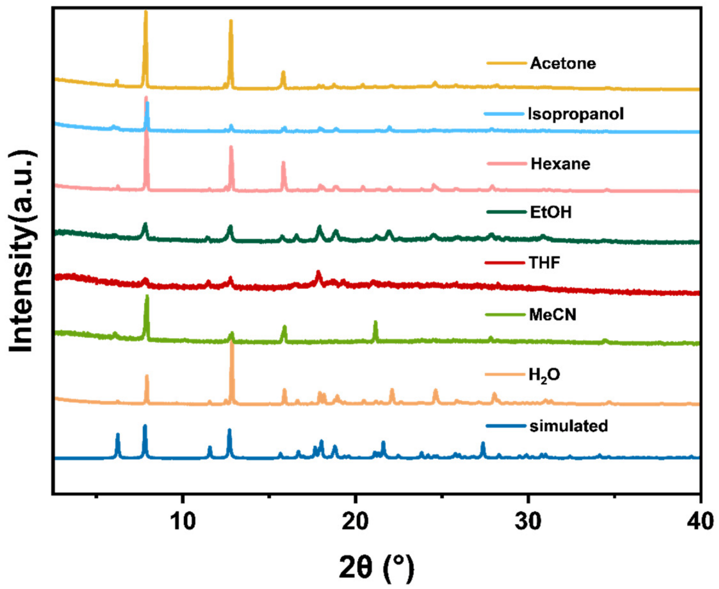

Figure S3. PXRD patterns of FDU-HOF-21 after treatment with different solvents for 24h.

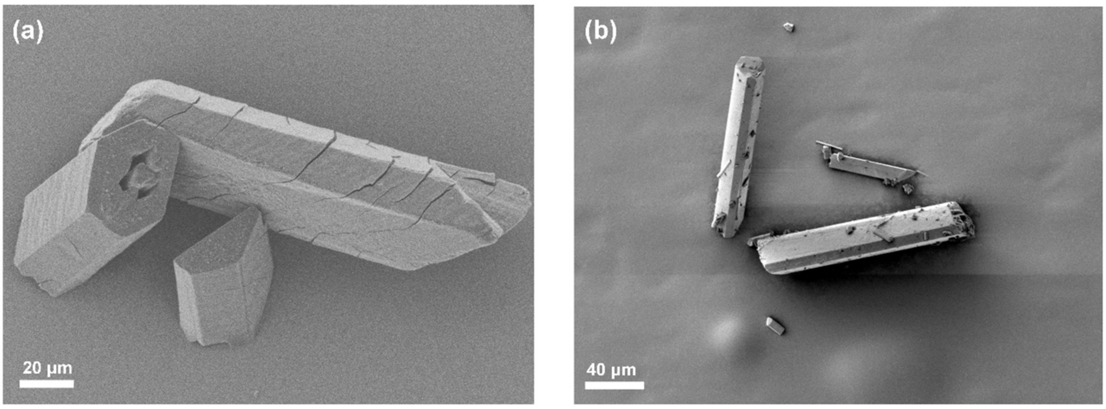

Figure S4. SEM images of FDU-HOF-21.

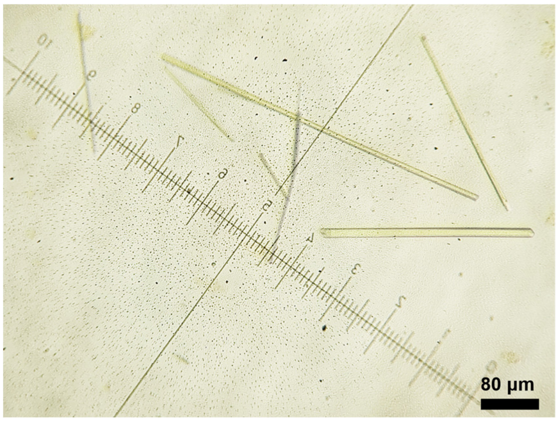

Figure S5. Microscopic optical photo of FDU-HOF-21.

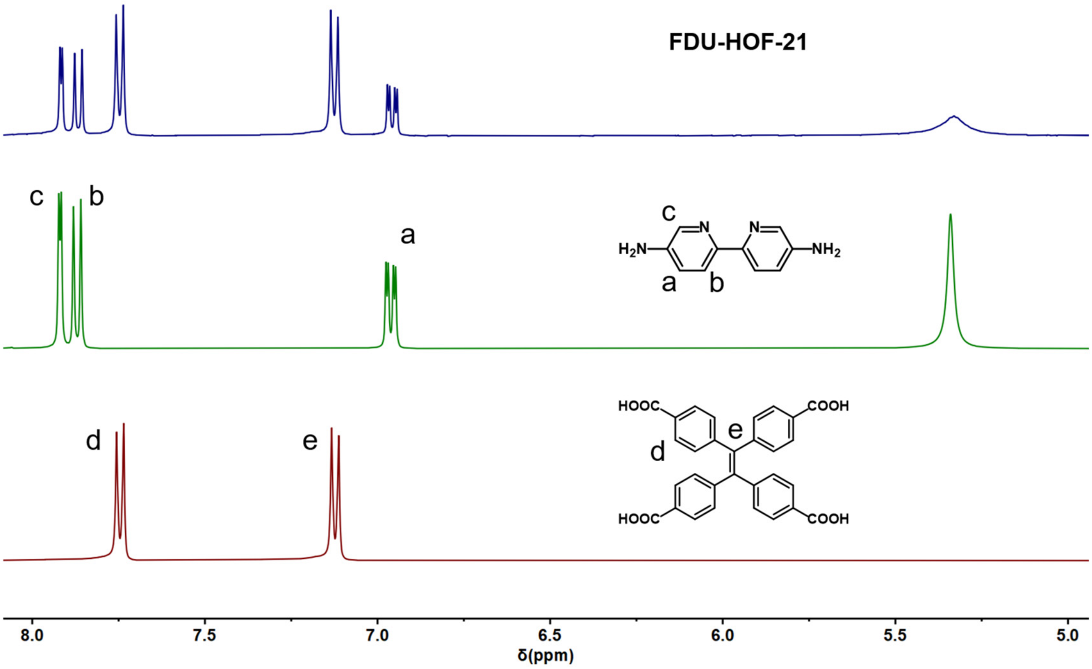

Figure S6. ^{1}H NMR spectra of FDU-HOF-21, NH_2Bpy and H_4TCPE (400 MHz, DMSO-d_6).

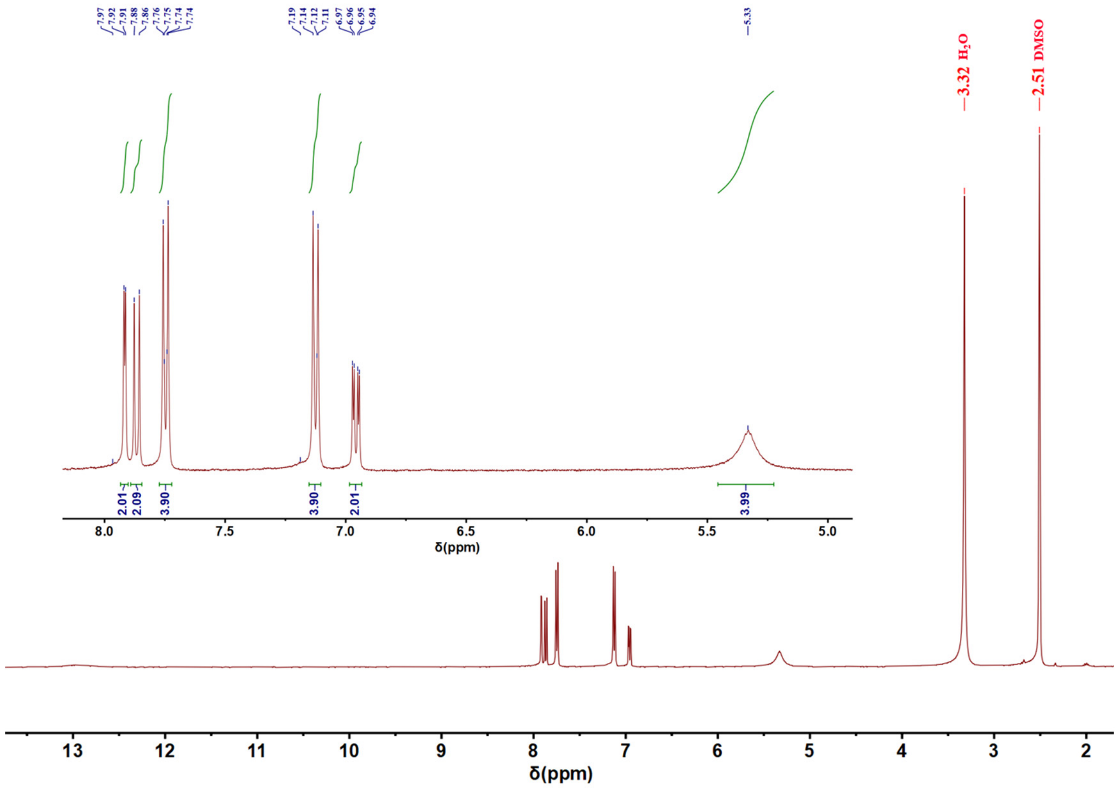

Figure S7. ^{1}H NMR spectra of FDU-HOF-21 and the corresponding integrations (400 MHz, DMSO-d_6).

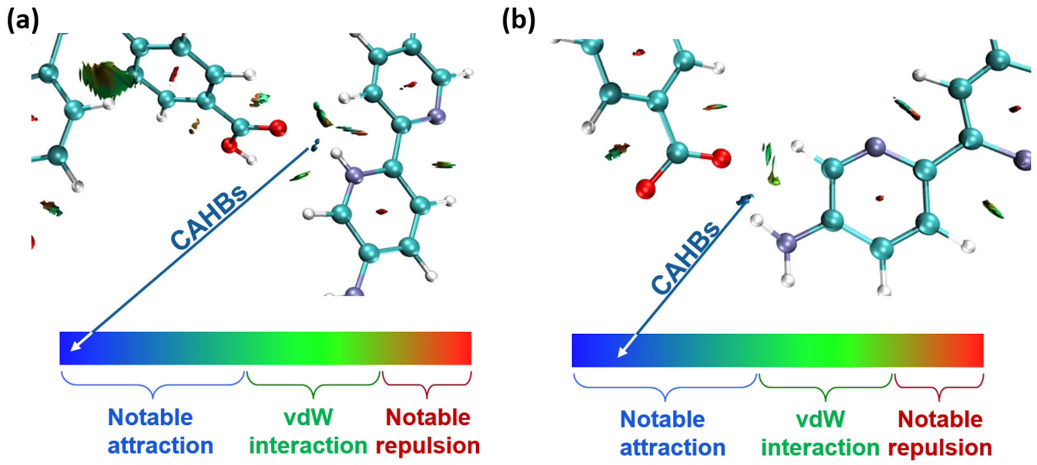

Figure S8. IRI analysis for FDU-HOF-21: (a) carboxylic acid–pyridine and (b) carboxylic acid–amino interactions. Red, green, and blue areas denote strong repulsion, van der Waals interactions, and strong attractive forces (CAHBs), respectively.

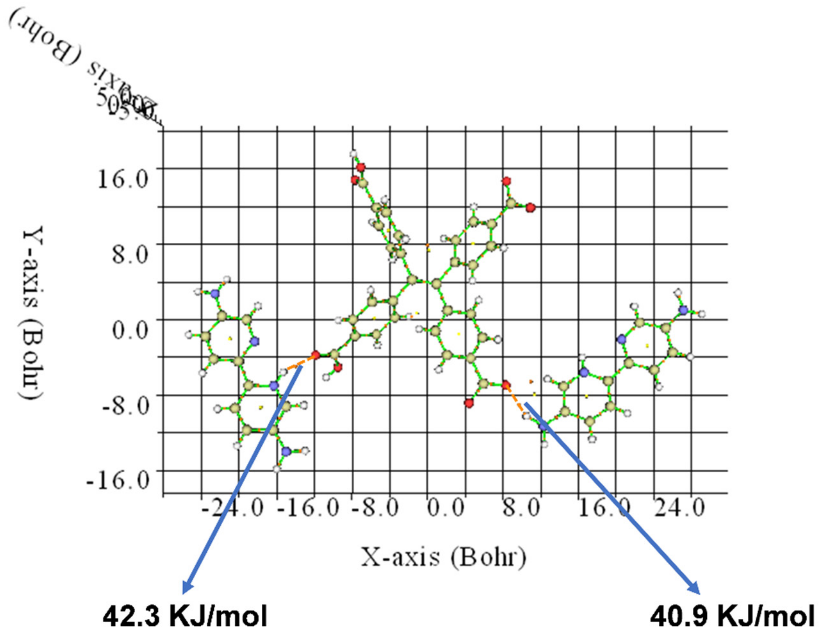

H-bond donor	H-bond Acceptor	bond length (Å)	bond energy (kJ/mol)	BCP
Pyridine N	O	2.715	-42.3	0.02720704 471
Amino N	O	2.960	-40.9	0.02621468 310

Figure S9. Calculation of the binding energies of the CAHBs.

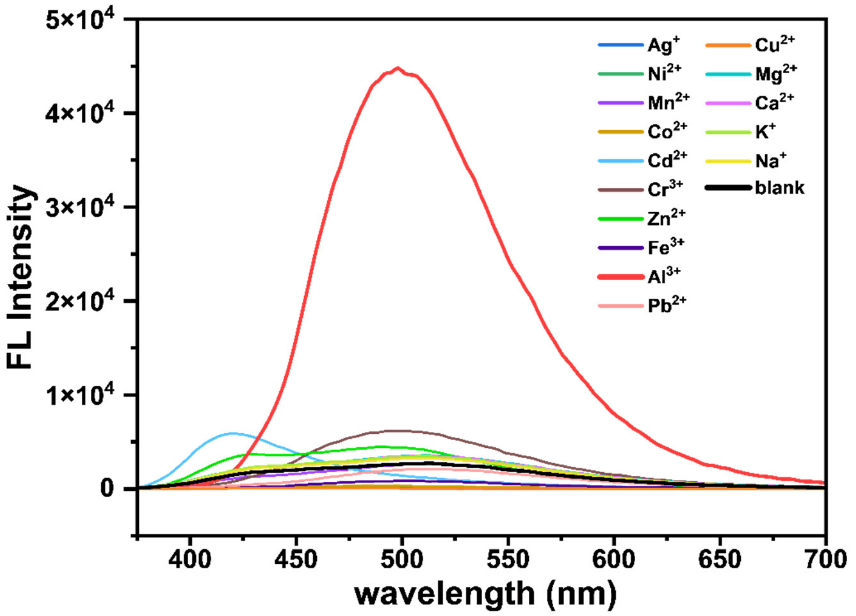

Figure S10. Fluorescence spectra of FDU-HOF-21 responded to different metal ions at λ_{ex} = 330 nm.

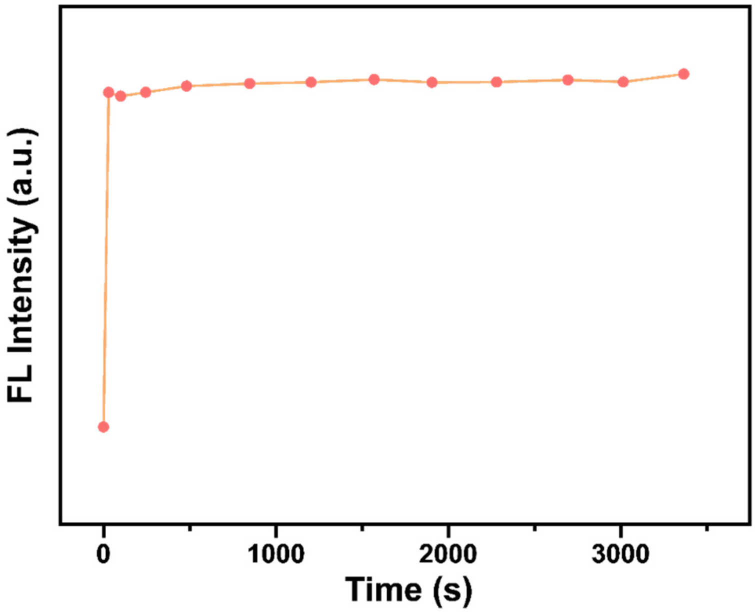

Figure S11. Fluorescence intensity of FDU-HOF-21 versus time at 330 nm upon addition of Al^{3+}.

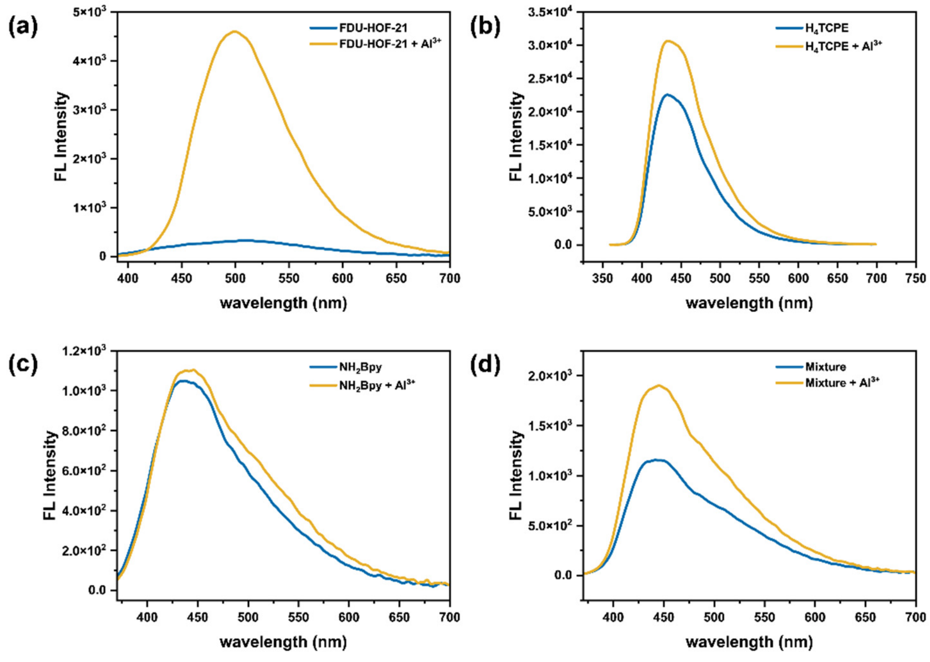

Figure S12. Fluorescence spectra of FDU-HOF-21, H_4TCPE, NH_2Bpy and H_4TCPE-NH_2Bpy mixture before and after responded to Al^{3+} ions.

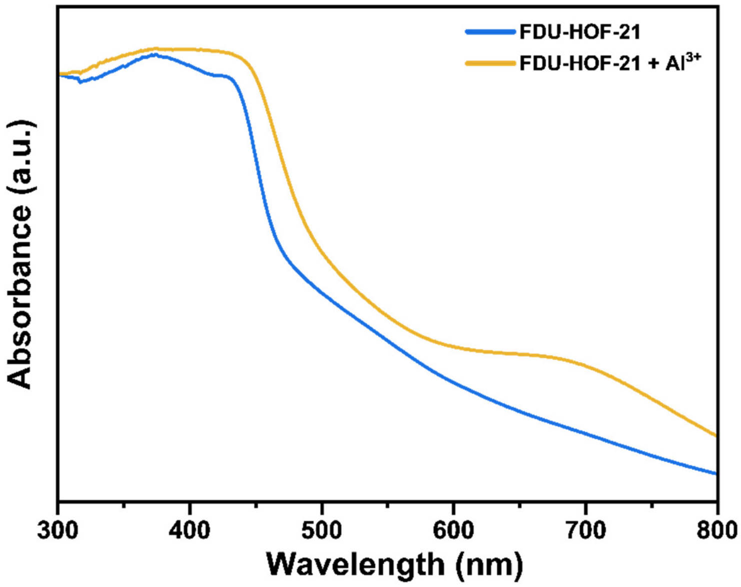

Figure S13. Solid-state UV-Vis spectra of FDU-HOF-21 before and after Al^{3+} binding.

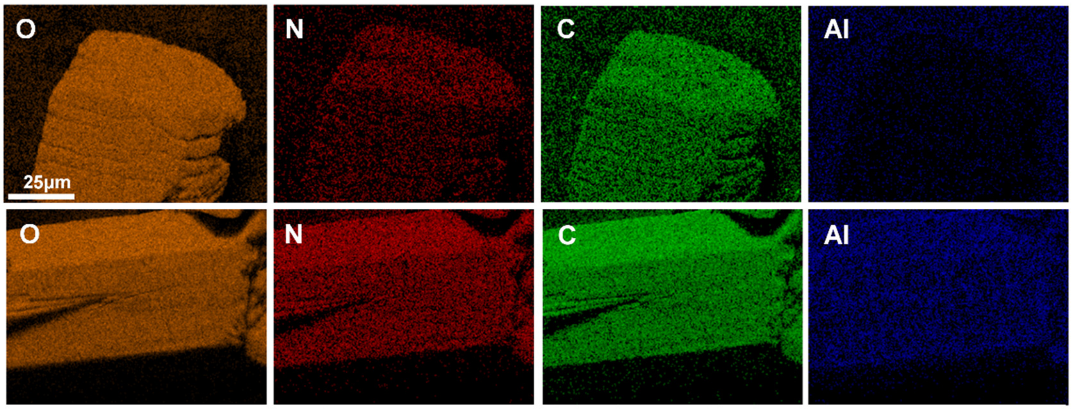

Figure S14. SEM-EDS elemental mapping images of FDU-HOF-21 for C, N, O, and Al (Top: before Al^{3+} binding; Bottom: after Al^{3+} binding).

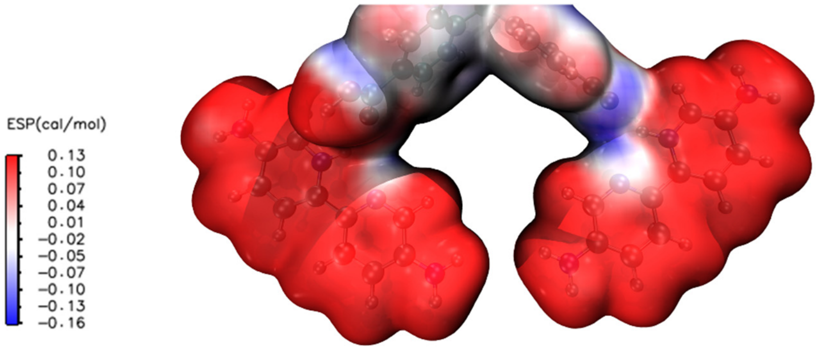

Figure S15. Electrostatic potentials mapped of FDU-HOF-21.

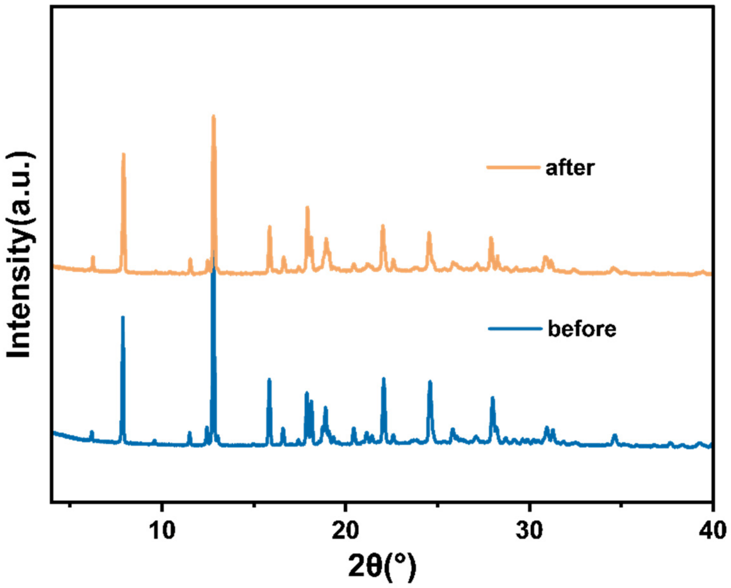

Figure S16. PXRD patterns of FDU-HOF-21 before and after binding with aluminum ions.

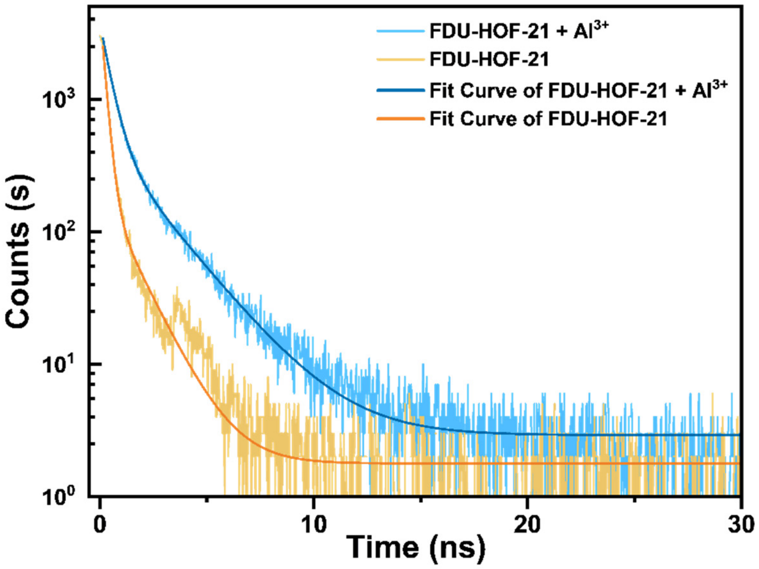

Figure S17. Time-resolved decay curves of FDU-HOF-21 before and after treatment by aluminum at λ_{ex} = 350 nm.

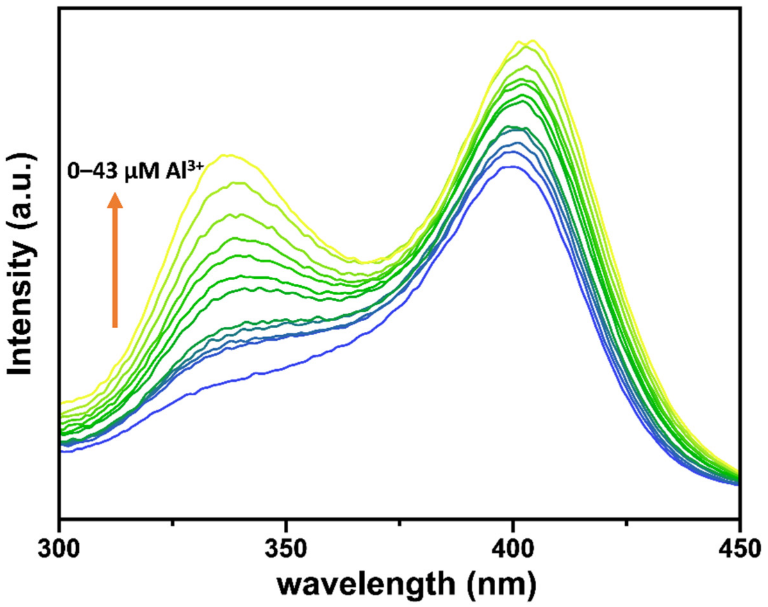

Figure S18. Fluorescence excitation spectra at λ_{em} = 510 nm of FDU-HOF-21 treated with different concentrations of Al^{3+} ions.
